# Admissibility Investigation and Validation of Infertility Distress Scale (IDS) in Iranian Infertile Women

**Published:** 2012-06-19

**Authors:** Khadijeh Arab-sheybani, Masoud Janbozorgi, Aygul Akyuz

**Affiliations:** 1 Department of Psychology, Payame Noor University, Tehran, Iran; 2 Department of Psychology, Research Institute of Hawzeh and University, Qom, Iran; 3 Department of Obstetrics and Gynaecologic Nursing, Nursing School, Gulhane Military Medical Academy, Ankara, Turkey

**Keywords:** Infertility, Validity, Women

## Abstract

**Background:**

Psychological stress has a profound effect on infertility and its treatment. The aim of this study was to develop a specific scale to determine distress levels among Iranian infertile women.

**Materials and Methods:**

In this cross-sectional study the samples included 300 women (145 fertile and 155 infertile) who completed the Infertility Distress Scale (IDS) form. Data was analysed using correlation method and main component analysis.

**Results:**

These results show that all 21 items had a high correlation with the overall scale. Cronbach’s alpha value was 0.91 for the entire list. Factor analysis results with 5 element extraction could identify 88% of overall variance with a special value higher than 1.

**Conclusion:**

According to results, the IDS questionnaire has enough admissibility and va- lidity in the measurement of the infertility distress scale in Iranian infertile women.

## Introduction

Infertility is defined as a failure to achieve pregnancy after one year of regular intercourse without preventive methods (1-3). This is one of the bitter experiences in life (2) which can be compared with a close relative death, and 10-15% of couples experience it (3-5). In other words, it represents a real life crisis which threatens couples’ psychological well-being (6-9). Thus infertile couples try to cope with treatment problems and infertility stress (10-12). Infertility stress is a group of signs which appear after infertility. It is like many other post-traumatic stress disorders (PTSD) and is especially evident in thoughts and feelings related to infertility and seeking to avoid it.

Problems in sleep, work, relations (especially marital relations), and painful sensitivity to any natural motives related to reproduction are observed in infertile people (13).

Although at the first stage infertility is a clinical condition, diagnosis can have many effects on the psychological performance of couples. Moreover, the psychological dimension of infertility affects other aspects of couple’s personal lives including social and economic ones (14). Each couple may react differently to the problem and its treatment. However, anger, reduced self-esteem, communication problems, less life satisfaction, anxiety and depression are among the most common reactions (6, 7, 9, 11). In addition it affects the couple’s sexual relations (6, 15, 16).

Previous studies show that stress, anxiety and depression scales were significantly higher in infertile couples than in fertile couples (8, 10, 16). In this respect, women who are raised to be mothers in the future are affected more (8, 12, 14, 17). Their goal is to have a child and when this goal is not achieved, they feel unhealthy, unhappy and guilty (18). Pasch et al. have reported different depressive problems between infertile men and women (15). Women are more affected than men and suffer from anxiety, depression, anger, disappointment, weakness and lack of control (8, 10, 12, 17).

Investigations show that after the first year of marriage there is a lot of pressure on couples to have a child. This pressure increases during the third and fourth years (18). The most important goal in infertility treatment is supporting the couples to have a child, but in this respect the couple’s health should be a priority (10, 19). Therefore, considering the couple’s psychological state during diagnosis and treatment is a vital issue, as today infertility is related to the couple, not just the wife or husband (20).

In previous studies, different scales have been used to measure stress and depression in infertile women (7, 11, 10, 21, 22), one of which is Petrson and Newton Infertility Distress Questionnaire (23). This questionnaire is a multidimensional tool designed in London’s Health Sciences Centre. It considers the stress of infertile people in five dimensions: social, sexual, relations, life style without a child and the need to be a parent. Akyuz designed the Infertility Distress Scale in Turkey in 2008 (24). The tests were designed in three main phases: In the first stage some questions were made with respect to research on infertile women’s concerns. In the second stage, the questionnaires were reformed and a pre-test was carried with a group consisting of 20 samples (10 fertile, 10 infertile). Finally in the third stage, admissibility and validity of tests was confirmed in a group of 300 samples (155 infertile, 145 fertile). Cronbach’s alpha value was found as 0.933 in the study.

However, there is no tool to show all the problems and concerns of infertile women. Especially there is no admissible and valid scale in Iran and there are no proper tools to do efficient research in this field. Moreover due to a lack of technical tools, most of infertility investigations consider personal features, anxiety and depression using various standard tools. So an infertility depression questionnaire is a vital tool to do research in this field. The present article aims to evaluate valid and admissible tools which measure distress scale in Iranian women during infertility and treatment.

## Materials and Methods

### Participants

In this cross-sectional project, the study population included all infertile women referring to Yazd infertility centre and all fertile women in Yazd. 155 infertile women within the age range of 22 to 41 years (mean age 29.1 years) and 145 fertile women with mean age of 30.2 years were chosen as a sample. From 2010, all women referring to Yazd infertility centre who had been married for at least two years, had no children, had been diagnosed as infertile by a physician and did not have any treatment experiences filled in the Infertility Distress Scale questionnaire and Fertility Problem Inventory. Prior to questionnaire completion, first they signed the consent form, the aim of the research was explained and they filled it with their own consent. Participants were informed that this research did not have any impact on the treatment process and they could give it back. Cluster sampling was used for fertile women. One region was chosen from three regions in Yazd and then 145 mothers were selected as a fertile sample from one of the schools.

### Tools

#### Infertility Distress Scale (IDS)

This questionnaire includes 21 multiple choice items which are scaled from 1 to 4, except 5 questions with opposite scaling from 4 to 1. The total scale of 21 items made the overall scale range from 21 to 84. To apply the questionnaire on Iranian participants, first it was received from Akyuz et al. (24) and translated into Persian. To be more valid, 6 professors in Shahid Beheshti and Yazd Medical Sciences Universities studied and reformed it. Finally participants were asked to read each item carefully and choose the sentence which best described their feelings.

#### Fertility Problem Inventory

This questionnaire consists of 46 questions and considers infertility in 5 dimensions: social, sexual, relations, life style without a child and the need to be a parent (23). In this method, internal correlation was 0.87 for social issues, 0.77 sexual issues, 0.82 relations, 0.80 life style without a child, 0.84 need to be a parent, and 0.93 overall stress. In Alizadeh and colleagues’ research (14) which was carried out on a sample of 30 infertile women, Cronbach’s alpha value was 0.78 for social issues, 0.77 sexual, 0.78 relations, 0.75 life style without a child, 0.84 need to be a parent and 0.91 overall stresses.

Data was analysed using exploratory factor analysis, Bartlett test of sphericity and Varimax rotation.

This study was approved by the Ethics Committee of the Institutional Review Board of Payam-e-Noor University of Southern khorasan province.

## Results

Cronbach’s alpha value, re-test and parallel forms were used for the validation and reliability measurement of this questionnaire.

Table 1 presents statistical features for 21 items, overall scale, every item’s correlation to overall scale and the effects of deleting every item on Cronbach’s alpha value on participants. Table 2 is for infertile samples.

It presents statistical features for 21 items, overall scale, every item’s correlation to overall scale and the effects of deleting every item on Cronbach’s alpha value on participants. The average of 21 items was 3.153 (item 18) -1.086 (item 12) (Table 1).

As is shown in table 2, the average of 21 items was 1.148 (item 12) - 3.961 (item 14) with standard deviation 0.427 (item 15) -2.440 (item 13). Average and overall scale standard deviation were 59.096 and 12.738, respectively. Cronbach’s alpha value was 0.91 for the overall scale and did not show a meaningful increase after deleting items, so it is not necessary to delete the experiment’s items (Tables1, 2). Moreover, this alpha showed correlation coefficient 0.89 for 30 people through re-experimentation of 80 participants after a 4-month interval, which was meaningful at 0.001 levels. In final consideration by parallel forms, 50 infertile participants were studied using Petrson and Newton (23) Infertility Questionnaire with Alizadeh and colleagues (14) Cronbach’s alpha value which was 0.91. As a result correlation coefficient 0.75 was found.

**Table 1:** Average, standard deviation and correlation of items to overall scale and Cronbach’s alpha value in case of deleting an item (n=300)


Item	M	SD	Correlationwith totalscore	Cronbach ’salpha if itemdeleted

**Question1**	2.063	0.740	0.809	0.901
**Question 2**	2.330	0.842	0.531	0.905
**Question 3**	3.146	1.237	0.188	0.614
**Question 4**	2.070	0.856	0.463	0.907
**Question 5**	2.163	1.160	0.646	0.902
**Question 6**	2.163	1.160	0.646	0.902
**Question 7**	2.586	1.131	0.465	0.907
**Question 8**	2.430	0.766	0.755	0.902
**Question 9**	2.246	0.977	0.258	0.911
**Question 10**	2.953	1.120	0.387	0.909
**Question 11**	1.773	1.194	0.741	0.900
**Question 12**	1.086	0.431	0.197	0.910
**Question 13**	2.000	1.416	0.833	0.869
**Question 14**	3.980	0.244	0.072	0.912
**Question 15**	2.020	1.423	0.839	0.869
**Question 16**	3.026	1.229	0.526	0.905
**Question 17**	2.513	1.045	0.646	0.902
**Question18**	3.153	1.270	0.413	0.903
**Question19**	2.563	0.924	0.773	0.900
**Question20**	2.513	1.045	0.646	0.902
**Question21**	2.463	1.371	0.366	0.911


M; Mean. SD; Standard deviation.

**Table 2:** Average, standard deviation and correlation of items to overall scale and Cronbach’s alpha in case of deleting an item (n=155)


Item	M	SD	Correlationwith totalscore	Cronbach ’salpha if itemdeleted
**Question1**	2.395	0.801	0.715	0.898
**Question 2**	2.535	0.808	0.499	0.902
**Question 3**	3.535	0.982	0.360	0.905
**Question 4**	2.303	0.767	0.469	0.902
**Question 5**	2.612	1.186	0.578	0.900
**Question 6**	2.612	1.186	0.578	0.900
**Question 7**	2.768	1.086	0.341	0.906
**Question 8**	2.858	0.784	0.785	0.898
**Question 9**	2.329	1.195	0.135	0.912
**Question 10**	3.458	0.961	0.583	0.900
**Question 11**	2.496	1.926	0.700	0.897
**Question 12**	1.148	0.544	0.145	0.908
**Question 13**	2.935	1.440	0.875	0.890
**Question 14**	3.961	0.339	0.350	0.909
**Question 15**	2.974	1.427	0.887	0.890
**Question 16**	3.180	0.942	0.454	0.903
**Question 17**	2.767	1.049	0.526	0.901
**Question18**	3.425	0.993	0.538	0.901
**Question19**	2.864	0.765	0.744	0.898
**Question20**	2.767	1.049	0.526	0.901
**Question21**	3.167	1.027	0.587	0.900


M; Mean. SD; Standard deviation.

Correlation coefficient between IDS and special variables is presented in table 3.

**Table 3:** T-test to consider IDS difference in fertile and infertile groups


Group	N	SD	M	T- test	P value

**Infertile **	155	12.738	-13.205	0.001	
**Fertile**	145	7.812	42.855		


M; Mean. SD; Standard deviation.

Conceptual admissibility of the questionnaire was confirmed by 10 experts (psychologist, psychiatrist, women’s expert). With respect to the factor analysis method, main component analysis and Varimax rotation were used. Moreover because the basic goal of factor analysis is to limit a lot of variables to a limited number of factors with the least rate of data lost, exploration of factor analysis was considered useful here. This method is used when a researcher does not have enough evidence to make an assumption about the number of fundamental elements. In fact this method uses data to justify the relation between variables (25) and the sample needed is 10-20 times bigger than the items (26).

According to data analysis, the results for Kaiser-Meyer-Olkin (KMO) were higher than 0.5 and the level of meaningfulness in the Bartlett Test of Sphericity was less than 0.05, so present data can be used as factors (25). Special scales were higher than 1 for factor analysis according to which 5 factors were extracted. Diagram and table 5 show factors, special scales, variance percentage, explanatory percentage, and explanatory variance accumulative percentage.

**Fig 1:**
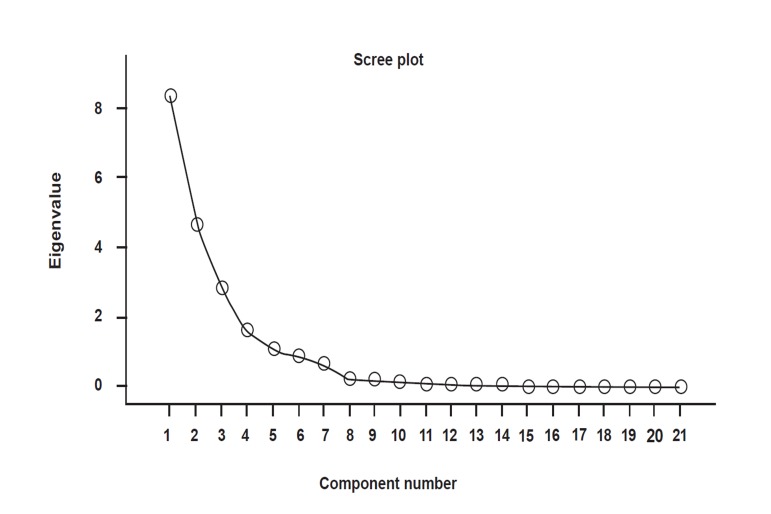
Rock diagram for identifying infertility depression scale.

The horizontal axis shows the items and the vertical axis represents special scales. According to figure 1 and tables 4-5, factor analysis also confirms infertility depression scale admissibility. Table 5 shows a meaningful correlation between Varimax rotation and factor load higher than 1 for 21 items with 5 factors. This method is used here because it produces factors with strong correlation between a small group of variables and another group with a weak correlation between variables.

Moreover variance maximizes the square of factor loads in each column and minimizes the number of variables with a strong load in each factor.

Because factor loads between 0.2-0.5 are proper for a question in every factor (26), the present article uses special amounts higher than 0.46 to choose the items of every factor. According to Table 4, five elements were extracted to identify 88.464% of overall variance.

**Table 4:** Variance percentage, variance accumulative percentage and special scales for 5 factors


Component	Total	Of variance (%)	Cumulative (%)
			

**1**	8.391	39.955	39.955
**2**	4.689	22.327	62.282
**3**	2.811	13.387	75.670
**4**	1.627	7.748	83.418
**5**	1.060	5.046	88.464


**Table 5:** Infertility depression test, rotated factor matrix in Varimax method


Item	Component				
	1	2	3	4	5

**1. I feel as if I were alone in the world**	0.843				
**2. I feel myself excluded out of my familyand friends**	0.589				
**5. I feel myself useless**	0.729				
**6. I feel myself unhealthy**	0.729				
**8. I have no pleasure from any of my works**	0.750				
**11. I avoid to talk about not being able tohave a child**	0.730				
**15.I think people around me accuse me ofnot being able to have a child**	0.809				
**17. That I cannot have a child affects**	0.736				
**sexual partnership with my husband**					
**18.I feel anger to my husband**	0.730				
**19.I think my husband does not currentlylove me as much as previously**	0.844				
**20.Relationship between me and myhusband has been affected negatively**	0.736				
**7. I feel myself anxious and nervouscontinuously**		0.565			
**10.I much more take care of myself whencompared to previous time**		0.762			
**13.My husband and I easily talk aboutnot being able to have a child**		0.542			
**16.I think my husband accuse me**		0.645			
**21.My husband is interested in me muchmore than before**		0.858			
**3. There are people around me to whom Ican admit when I am bored**			0.703		
**4. I have no more power to resist andstruggle**			0.797		
**9. I feel myself continuously tired recently**				0.650	
**14.I easily have friendship with familieswho have children**					0.569
**12.I would not like being asked questionsabout not being able to have a child**					0.777


## Discussion

Validity investigation by Cronbach’s alpha value confirmed the same role of all items in overall scale, and item deletion did not increase alpha. Therefore, it was not necessary to delete or change items. Cronbach’s alpha values were 0.76 and 0.091 for fertile and infertile women, respectively. Moreover with respect to the t test there was a meaningful difference in IDS scale for these two groups. This is due to internal pressures and the need to be a mother and also social pressures because of not having a child which increases stress in infertile women. In other words, one can mention cultural elements, i.e; the role of mother is considered an important part of women’s existence in different societies. Different researches show that this is the most important and satisfying role for women (14). However if men are not successful in domestic roles they find other sources of satisfaction and can compensate for infertility through professional and social tasks. Moreover as Abbey’s studies show, a lot of experiments and treatments are done on women that can increase stress and decrease self-confidence (27). Another reason is that infertile women show more responsibility than men, with respect to infertility. In most societies women are held responsible for infertility which causes a feeling of guilt and decrease in self-esteem. Women develop their identity by child birth because it is a natural process and symbolizes maturity, adulthood and femininity (16). Infertility is not a biological crisis but it is the basis of an identity crisis (14).

On the other hand, infertility is related to women at first and failure to achieve pregnancy results in pressures from the husband’s family. Finally there is a concern that non-pregnancy is sufficient reason for divorce or husband’s re-marriage. Besides, infertile men are more depressed but women show the same levels depression regardless of whether they or their husbands are infertile. All the evidence shows that infertile women have more stress and depression than fertile women.

According to the results, higher educational level decreases the effects of infertility pressures and leads to lower scales in questionnaires. In this article, factor analysis method and correlation coefficient higher than 0.46 resulted in 5 factors with special value higher than 1, which identified 0.88% of overall variance. Cronbach’s alpha value 0.89 was also found by re-experimentation of 30 samples (15 fertile, 15 infertile). Therefore, this tool can be used to measure infertility depression scale in Iranian women and proper confrontation with this problem. The limited scale volume is one of the problems which needs careful generalisation to all society. It is hoped that using IDS in other research can help to achieve valuable results and decrease infertility depression. Moreover, proper education to confront infertility is the best way to reduce stress in this group.

## Conclusion

According to the results, the Infertility Distress Scale questionnaire has enough admissibility and validity in the measurement of the infertility distress scale in Iranian infertile women.

## References

[1] Hardeep L, Rohtash S, Bindu K (2009). Psychological Distress, coping and subjective wellbing among infertile women. J Academy of Applied psychology.

[2] van den Akker OB (2005). Coping, quality of life and psychosocial symptoms in three groups of sub- fertile women. Patient Educ Couns.

[3] Bharadwaj A, Van Balen F, Gerrits T, Inhorn M (2000). Infertility and gender: a perspective from India. Social science research on childlessness in a global perspective.

[4] Bovine J, Bunting L, Collins JA, Negron KG (2007). International estimates of infertility prevalence and treatment-seeking: potential need and demand for infertility care. Hum Reprod.

[5] Van Balen F, Gerrits T (2001). Quality of infertility care in poor-resource areas and the introduction of new reproductive technologies. Hum Reprod.

[6] Cooper BC, Gerber JR, McGettrick AL, Johnson JV (2007). Perceived infertility-realted stress correlates with in vitro fertilization outcome. Fertil Steril.

[7] Faramarzi M, Alipor A, Esmaelzadeh S, Kheirkhah F, Poladi K, Pash H (2008). Treatment of depression and anxiety in infertile women: cognitive behavioral therapy versus fluoxetine. J Affec Disord.

[8] Mindes EJ, Ingram KM, Kliewer W, James CA (2003). Longitudinal analyses of the relationship between unsupportive social interactions and psychological adjustment among women with fertility problems. Soc Sci Med.

[9] Ramezanzadeh F, Aghssa MM, Abedinia N, Zayeri F, Khanafshar N, Shariat M (2004). A surveyof relationship between anxiety, depression and duration of infertility. BMC Womens Health.

[10] Matsubayashi H, Hosaka T, Izumi S, Suzuki T, Kondo A, Makino T (2004). Increased depression and anxiety in infertile Japanese women resulting from lack of husband’s support and feelings of stress. Gen Hosp Psychiatry.

[11] Wang K, Li J, Zhang JX, Zhang L, Yu J, Jiang P (2007). Psychological characteristics and marital quality of infertile women registered for in vitro fertilization-intracytoplasmic sperm injection in China. Fertil Steril.

[12] Cousineau TM, Green TC, Corsini EA, Barnard T, Seibring AR, Domar AD (2006). Development and validation of the Infertility Self-Efficacy scale. Fertil Steril.

[13] Joshi HL, Singh R (2009). Psychological distress, coping and subjective wellbeing among infertile women. J IAAP.

[14] Alizadeh T, Farahani MT, Shahr Aray M, Alizadegan S (2005). The relationship between self esteem and locus of control with stress in infertile women and men. J Reprod Fertil.

[15] Pasch LA, Dunkel-Schetter C, Christensen A (2002). Differences between husbands’ and wives’ approach to infertility affect marital communication and adjustment. Fertil Steril.

[16] Zorn B, Auger J, Velikonja V, Kolbezen M, Meden-Vrtovec H (2008). Psychological factors in male partners of infertile couples: relationship with semen quality and early miscarriage. Int J Androl.

[17] Gürhan N, Oflaz F, Atıcı D, Akyüz A, Vural G (2007). Effectiveness of nursing counseling on coping and depression in women undergoing in vitro fertilization. Psychol Rep.

[18] Benyamini Y, Gozlan M, Kokia E (2005). Variability in the difficulties experienced by women undergoing infertility treatments. Fertil Steril.

[19] Akyüz A, İnanc N, Pabuçcu R (1999). Determining the experiences and basic needs that guide nursing activities for the couples attending the IVF Unit. Gulhane Med J.

[20] Akyüz A (2001). Nursing in adaptation to IVF failure.Presented for the Ph.D..

[21] Sandelowski M (1999). Culture, conceptive technology, and nursing. Int J Nurs Stud.

[22] Beutel M, Kupfer J, Kirchmeyer P, Kehde S, Köhn FM, Schroeder-Printzen I (1999). Treatment-related stresses and depression in couples undergoing assisted reproductive treatment by IVF or ICSI. Andrologia.

[23] Peterson B (2003). Newton C.Examining congruence betweenpartner’ perceived infertility related stress and its relationshipto marital adjustment and depression in infertile couples. Fam Process.

[24] Akyuz A, Gurhan N, Baklr B (2008). Development and validation of an Infertility Distress Scale for Turkish Women. TAF Prev Med Bull.

[25] Kalantari KH (2006). Processing and data analysis in economical and social researches.

[26] Hooman HA (2002). Analyzied of multivariable data in research behaviour.

[27] Abbey F, Andrews M, Hallmant L (1992). Infertility and subjective well being: The mediating roles of self esteem, internal control and interpersonal conflict. J Marriage Fam.

